# No wonder, it is a hybrid. Natural hybridization between *Jacobaea vulgaris* and *J. erucifolia* revealed by molecular marker systems and its potential ecological impact

**DOI:** 10.1002/ece3.10467

**Published:** 2023-08-30

**Authors:** Barbara Gawrońska, Małgorzata Marszałek, Piotr Kosiński, Marek Podsiedlik, Leszek Bednorz, Joanna Zeyland

**Affiliations:** ^1^ Department of Biochemistry and Biotechnology, Faculty of Agronomy, Horticulture and Bioengineering Poznań University of Life Sciences Poznań Poland; ^2^ Department of Botany, Faculty of Agronomy, Horticulture and Bioengineering Poznań University of Life Sciences Poznań Poland; ^3^ Institute of Dendrology Polish Academy of Sciences Kórnik Poland; ^4^ Natural History Collections, Faculty of Biology Adam Mickiewicz University in Poznań Poznań Poland

**Keywords:** admixture, amplified fragment length polymorphism (AFLP), cpDNA, hybridization, *Jacobaea erucifolia*, *Jacobaea vulgaris*

## Abstract

Progressive changes in the environment are related to modifications of the habitat. Introducing exotic species, and interbreeding between species can lead to processes that in the case of rare species or small populations threatens their integrity. Given the declining trends of many populations due to increased hybridization, early recognition of hybrids becomes important in conservation management. Natural hybridization is prevalent in *Jacobaea*. There are many naturally occurring interspecific hybrids in this genus, including those between *Jacobaea vulgaris* and its relatives. Although *Jacobaea erucifolia* and *J. vulgaris* often co‐occur and are considered closely related, apart from the few reports of German botanists on the existence of such hybrids, there is no information on research confirming hybridization between them. Morphologically intermediate individuals, found in the sympatric distributions of *J. vulgaris* and *J. erucifolia*, were hypothesized to be their hybrids. Two molecular marker systems (nuclear and chloroplast DNA markers) were employed to test this hypothesis and characterize putative hybrids. Nuclear and chloroplast DNA sequencing results and taxon‐specific amplified fragment length polymorphism (AFLP) fragment distribution analysis confirmed the hybrid nature of all 25 putative hybrids. The AFLP patterns of most hybrids demonstrated a closer relationship to *J. erucifolia*, suggesting frequent backcrossing. Moreover, they showed that several individuals previously described as pure were probably also of hybrid origin, backcrosses to *J. erucifolia* and *J. vulgaris*. This study provides the first molecular confirmation that natural hybrids between *J. vulgaris* and *J. erucifolia* occur in Poland. Hybridization appeared to be bidirectional but asymmetrical with *J. vulgaris* as the usual maternal parent.

## Graphical abstract

Morphologically intermediate individuals, found in the sympatric distributions of *J. vulgaris* and *J. erucifolia*, were hypothesized to be their hybrids. Two molecular marker systems were employed to test this hypothesis and characterize putative hybrids. This study provides the first molecular confirmation that natural hybrids between *J. vulgaris* and *J. erucifolia* occur in Poland. Hybridization appeared asymmetrical and bidirectional with *J. vulgaris* as the usual maternal parent.

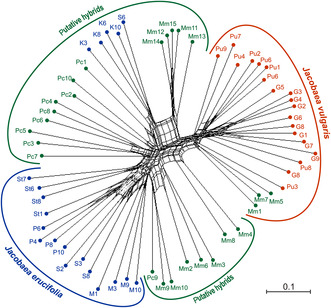

## 1 INTRODUCTION

Natural hybridization and introgression are widespread in the plant world (Grant, 1972; Mallet, 2001; Rieseberg et al., 2004), and their role is widely discussed in recent literature (Bush, 2022; Ebersbach et al., 2020; Hobbs et al., 2022; Nevado et al., 2020; Walter et al., 2020a; Wong et al., 2020). In the past decade, the use of a number of molecular methods, in particular whole‐genome sequencing, has made it possible to detect not only ongoing hybridization events, but also ancient hybridization events (Arantes et al., 2020; Chaturvedi et al., 2020; Geraldes et al., 2014; Moran et al., 2021; Salvatori et al., 2019; Taylor & Larson, 2019).

From a biological point of view, the hybridization process is important because, by increasing the genetic variety within a species, it determines evolution and may play a substantial role in the adaptation and speciation of plant populations (Barton, 2001; Rieseberg et al., 2003). Hybridization can occur both within and between taxa, leading to very different consequences, depending on species and environmental conditions. Moreover, hybridization may have either negative or positive impacts (Hegarty, 2012; Rieseberg, 1997; Schierenbeck & Ellstrand, 2009). The importance of natural hybridization in the process of speciation, that is in the creation of new biodiversity, was first pointed out by botanists (Abbott, 1992; Abbott et al., 2010, 2013; Abbott & Rieseberg, 2012; Fitzpatrick et al., 2009; Mallet, 2007; Nosil et al., 2009). Hybridization can lead to the formation of new species and new adaptive traits that enable them to occupy new niches and expand their range (Hamilton & Miller, 2016; Rieseberg et al., 2003). On the other hand, it may also lead to the replacement of native species by invasive species (pure species can become maladaptive; Guo, 2014; Huxel, 1999), which may result in loss of biodiversity. This is important in the context of climate changes, which cause changes in species distribution (Brennan et al., 2014; Walther, 2010). In addition, due to increasing global changes, the rate of hybridization seems to be increasing (Brennan et al., 2014; Pfennig et al., 2016).

Recent studies have shown that hybridization can have more complex outcomes. One of the open questions concerns debate whether hybrids are an evolutionary dead end or play a more creative role in adaptation and speciation (Anderson, 1948; Rieseberg, 1997). As mentioned above, hybridization plays a large role in speciation, and even if it does not lead to speciation, it can provide opportunities for adaptation (Wong et al., 2022). However, hybridization is often considered a dead end because some fraction of hybrid individuals never reproduces (they are not fully fertile). Even when they are fertile, hybrids are usually reabsorbed into the population of one or other parental species via backcrossing. Molecular data revealed that species described and long suspected to be a homoploid hybrid species (such as the long‐lived *Juglans hopeiensis*, *Athrotaxis laxifolia*, and *Actinidia zhejiangensis*) actually consist mainly of first‐generation hybrids and advanced generation backcrosses. Despite their distinct morphological traits and long recurrent hybrid origination, they do not initiate speciation. (Worth et al., 2016; Yu et al., 2023; Zhang et al., 2021). These cases highlight that conclusions about the large role of hybridization in speciation, at least as regards homoploid hybrids, as well as the time needed for their reproductive isolation, require further research. On the one hand, hybrids have to overcome two main challenges related to hybrid infertility and backcrossing with parental lines (Linder & Rieseberg, 2004), on the other hand, sterility is a mechanism of post‐zygotic isolation as a consequence of various processes of genetic or chromosomal origin (Stebbins, 1958; Yakimowski & Rieseberg, 2014). In the absence of reproductive isolation, hybridization can lead to a reduction or loss of differentiation in the process termed introgressive hybridization (Abbott et al., 2013; Anderson, 1949). In this case, backcrossing of hybrids with one or both parental species leads to genetic erosion of the parental gene pools (Carnicero et al., 2023; Linder & Rieseberg, 2004). Typically, introgression refers to the flow of alleles/genes by repeated backcrossing of interspecific hybrids with the parental species. It is a natural evolutionary process that can have positive impacts on biodiversity. However, it can also be a source of genetic erosion (Allendorf et al., 2001). The interbreeding of populations that were formerly isolated from each other can impair the genetic integrity of either or both populations when hybridization progresses to introgression. In the case of local endemic species and small isolated populations, introgressive hybridization may therefore lead to a serious threat.

According to Walter et al. (2020b), genetic interactions created by recombination among derived alleles from the divergent populations could have positive (e.g., adaptive introgression) or negative (e.g., genetic incompatibilities) effects on fitness. It remains largely unknown whether these same advantageous alleles which accumulate in each population, during the process of adaptation to contrasting environments, create genetic incompatibilities that can cause intrinsic reproductive isolation leading to speciation. Moreover, dominant alleles can create heterosis in hybrids (Brennan et al., 2014; Wong et al., 2022). An example of heterosis was described in the F1 hybrids between *Jacobaea vulgaris* and *J. aquatica* (Kirk, Vrieling, & Klinkhamer, 2005).

Hybrid incompatibility frequently results from hybridization between more divergent taxa (Abbott et al., 2013; Moyle & Nakazato, 2010; Zhang et al., 2021). Besides multilocus Bateson–Dobzhansky–Muller (BDM) incompatibilities involving interactions between nuclear genes or between nuclear and organellar genes, as another kind of important postzygotic isolating barrier in plants, chromosomal rearrangements have been recognized (Baack et al., 2015). According to Rieseberg (2001), they can directly affect hybrid fitness or also may increase the strength of genic barriers. Negative nuclear genetic interactions were largely the cause of reductions in hybrid fitness (Walter et al., 2020b).

Whereas in the case of natural hybridization, novel phenotypes may be formed, increasing genetic diversity at both the population and species levels, hybridization due to human activity may have the reverse effects because it may threaten the genetic integrity and persistence of native species. Human activities are one of the causes of hybridization (Anderson & Stebbins, 1954). However, the question arises to what extent hybridization can be caused or at least enhanced by human activity. For example, such activities may lead to contact between previously isolated taxa, making hybridization possible (Gawrońska et al., 2019). Other examples include “species introduction” (where non‐native species have been introduced by human activities) and “habitat disturbance” (where hybridization was enhanced by habitat disturbance; Todesco et al., 2016). Human activities promote hybridization, creating opportunities for its occurrence. Environmental changes may increase the fitness of hybrids relative to parental genotypes, create new niches that are favorable to hybrid genotypes and thus cause the extinction of native species (Guo, 2014).

In light of this information, hybridization, when motivated by anthropogenic factors, can be considered as one of the significant problems for conservation. In a world that is rapidly changing under the influence of human activity, maintaining the existing biodiversity becomes a major challenge (Allendorf et al., 2001; Brennan et al., 2014; Todesco et al., 2016). Climate change, modification of natural habitats, and the movement of invasive species threaten biodiversity (Todesco et al., 2016). Moreover, environmental changes and the introduction of exotic species can, through interbreeding between species, lead to processes known as demographic or genetic swamping, that threaten the integrity of small populations or rare species (Quilodran et al., 2020; Todesco et al., 2016). The risk of extinction of plant and animal taxa has already increased, and breaking of existing reproductive barriers between organisms may also disrupt their independent evolution (Quilodran et al., 2015, 2020). According to Todesco et al. (2016), “understanding the factors that contribute to destructive versus constructive outcomes of hybridization is key to managing conservation concerns”. At the same time, as management priorities, the authors have identified stopping the introduction of exotic species that are prone to hybridization and restoring mature and diverse habitats that are more resistant to hybridization and to the establishment of hybrid genotypes (Todesco et al., 2016).

Generally, we can say that hybridization adds problems (loss of biodiversity, ecosystem degradation) as well as solutions (new adaptive variation, ecosystem robustness) to global change challenges (Abbott et al., 2013; Brennan et al., 2014). However, it can also drive the emergence of new biodiversity, enhancing the ability to adapt to changing environmental conditions and stimulating the appearance of new species (Quilodran et al., 2020). Finally, hybridization can lead to adaptive divergence between populations and give rise to new populations of mixed ancestry that remain distinct from both parental populations (Abbott et al., 2010; Mallet, 2007). Understanding how biodiversity is distributed and maintained is closely related to explaining the evolution of the geographic ranges of species, which is especially important today in the face of ongoing biodiversity loss and global change (Pfennig et al., 2016). Although impressive progress has been made in documenting the extent of natural hybridization, both historical and recent, there are still many unanswered questions regarding the role of hybridization in the context of human activity and global changes in the world. According to some authors, hybridization may still have other unidentified ecological and evolutionary consequences (Guo, 2014; Orians, 2000).

Senecioneae is the largest tribe in the family Asteraceae, which covers over 160 genera with more than 3000 species, and new genera are still being added (Chen et al., 2011; Nordenstam et al., 2009). The tribe is remarkable for its size, rich morphological and ecological diversity, and nearly cosmopolitan distribution (Pelser et al., 2007). *Senecio* L. is the largest genus of this tribe, represented by 1250–1400 species (Nordenstam et al., 2009; Wong et al., 2022). In recent years, molecular phylogenetic studies have stimulated sectional and generic reorganization, and several genera have become more narrowly defined (Nordenstam et al., 2009; Pelser et al., 2007). Section *Jacobaea*, for example, previously being a part of the genus, was recently raised to the rank of the new genus *Jacobaea* Mill. (Pelser et al., 2004).

The role of hybridization in adaptation and speciation is well illustrated by numerous examples from different Senecioneae species. For this reason, they have become one of the popular plant models for studying the speciation process at the genetic, genomic, and ecological levels (Abbott & Rieseberg, 2012; Gross, 2012; Walter et al., 2020a; Wong et al., 2022).

*Jacobaea* species are known to hybridize in nature. Although several *Jacobaea* taxa are karyologically heterogeneous, having two or more ploidy levels (Hodalova et al., 2010, 2015; Liu et al., 2004), there are many (both within and across ploidy levels, between native and introduced taxa) naturally occurring or cultivated interspecific hybrids in this genus (e.g., Abbott et al., 2000, 2003, 2007, 2009; Abbott & Lowe, 2004; Chapman & Abbott, 2010; Comes & Abbott, 2001; James & Abbott, 2005; Kadereit et al., 2006; Kirk et al., 2004; Leiss, 2011; Lowe & Abbott, 2004; Pelser et al., 2012).

Many reports show numerous cases of introgressive hybridization between *J. vulgaris* and its relatives (e.g., Comes, 1994; Comes et al., 1997; Hodalova et al., 2002; Hodalova & Marhold, 1996). However, there is a lack of information on studies involving hybridization between *J. vulgaris* and *J. erucifolia*, although they often co‐occur and are considered closely related species due to similar morphological characteristics. To date, we can only find mentions of the presence of such hybrid under the name *Jacobaea* × *liechtensteinensis* (Murr) Rottenst (=*S*. × *whitwellianus* Murr) in Austria and Liechtenstein (Dinter, 1932; Hebbel, 2015/2016; Hegi, 1929).

*J. vulgaris* is a herbaceous, self‐incompatible, biennial, or short‐lived perennial plant that inhabits open, grassy (frequently disturbed) habitats throughout Europe and western Asia (Andersson, 2001). It is known to be an invasive species in several regions of the world, and due to its toxic secondary metabolites (e.g., pyrrolizidine alkaloids), it is an invasive and harmful weed in many countries (Kirk et al., 2004; Lin et al., 2019; Pelser et al., 2003). *J. vulgaris* is associated with waste places, sands, railways and roadsides (Hodalova et al., 2010, 2015). Generally, it is found in the drier regions; however, on other continents, *J. vulgaris* is noted in areas containing moderate moisture (for example, see Bain, 1991; Wardle, 1987). *J. vulgaris* is taxonomically heterogeneous, with four recognized subspecies. It is also an example of a karyologically variable species comprising five different cytotypes throughout Europe, with the most common occurrence of tetraploids (2*n* = 40; Hodalova et al., 2007, 2015).

*J. erucifolia* is a stoloniferous perennial plant and, like *J. vulgaris*, has a Eurasian distribution. However, unlike *J. vulgaris*, it is relatively rare in Poland and is scattered throughout the country, mainly in the southern part of Poland (Kruk & Sobisz, 2013). This species inhabits different habitats, but mainly ruderal sites (for review, see Podsiedlik & Bednorz, 2017). *J. erucifolia* is also taxonomically heterogeneous with five subspecies. Two of them, subsp. *erucifolia* and subsp. *tenuifolia*, are most commonly mentioned in European floras (Chater & Walters, 1976). Four presently existing Polish populations of *J. erucifolia* (Western Pomerania) represent subsp. *erucifolia*. The remaining populations found in the southern part of the country are currently classified as subspecies *tenuifolia* (Podsiedlik et al., 2016; Podsiedlik & Bednorz, 2017).

*J. vulgaris* and *J. erucifolia* occur in Poland, often in neighboring sites. Considering that closely related plant species frequently hybridize readily in contact zones, it is to be expected that this may also be the case here, especially as stated above, such putative hybrids, earlier identified based on morphology, have been reported. During field trips and while collecting material for studying the morphological diversity of the populations of *J. erucifolia* in Poland, in two stands where the two species coexisted, some morphologically intermediate individuals between *J. vulgaris* and *J. erucifolia* were found. These individuals were remarkably higher, with thicker and usually unbranched stems (as *J. vulgaris*), with the leaves with narrow lobes (as *J. erucifolia*) but not tucked up at the edges and covered with thicker hair layer. The underground part was a thick, horizontal root not resembling a typical rhizome of *J. erucifolia*. The individuals were found in the typical habitats for both species—abandoned farmlands and xerothermic grasslands.

Unambiguously, detection of introgressed genes in the offspring of a particular species based on morphological traits is difficult, and it may even be impossible. According to Allendorf et al. (2001), individuals from hybrid swarms that obtained most of their genes from one of the parental taxa are often morphologically indistinguishable from that parental taxon. Furthermore, plants often show a high degree of morphological plasticity in response to various environmental factors. In this case, the intermediate morphology may be due to factors other than hybridization, so morphological criteria alone for identifying natural hybrids or accurately determining the taxon are inadequate. Moreover, morphological variation could be relatively high within Senecioneae species and two morphologically similar species, *J. vulgaris* and *J. erucifolia*, are often confused (Pelser et al., 2002). In addition, *J. vulgaris* exhibits considerable variation in ligule morphology (Andersson, 2001). Now, molecular markers provide helpful tools to assess introgression in plant populations.

As mentioned above, morphological characteristics have limitations. In contrast, molecular markers based on DNA polymorphism are more informative, independent of environmental conditions, and abundant (Agarwal et al., 2008). However, it should be remembered that different types of markers carry information on other aspects of hybridization, then the identification of hybrid individuals requires the selection of an appropriate method in a given situation (Burgarella et al., 2009).

Among the most commonly used methods, there are analyses of organelle genomes (maternally inherited chloroplast and cytoplasmic mitochondrial markers), clustering‐based methods (fingerprinting – multilocus genotype data) such as amplified fragment length polymorphism (AFLP; Vos et al., 1995), and sequence comparison of nuclear ribosomal DNA internal transcribed spacers (ITSs) regions. This region has advantageous features such as biparental inheritance compared to the uniparental inheritance of organellar DNAs and the assumed intragenomic uniformity due to the active homogenization of repeat copies, known as concerted evolution (Nieto Feliner et al., 2017). These features make it the most frequently utilized and convenient target region for molecular identification of different organisms (Alvarez & Wendel, 2003). All these methods have already been applied to demonstrate hybridization and introgression and were successfully used in hybridization studies within different Senecioneae species (Kirk et al., 2004; Pelser et al., 2002, 2003, 2004; Raina et al., 2001; Rieseberg et al., 1995; Rieseberg & Wendel, 1993).

Initially, in this study, we aimed to test the hypothesis that specimens characterized by intermediate morphology are natural hybrids of two closely related *J. vulgaris* and *J. erucifolia* growing in Poland, using a combination of nuclear and plastid DNA sequence data and an AFLP. In addition, because *J. erucifolia* is a relatively rare species in Poland, the following questions arise: (1) Is it possible that native species are being displaced by hybrids, which threatens the extinction of pure parental genomes? (2) What consequences may result from the spread of hybrid individuals in the future? and (3) In the light of the reports on the impact of hybridization on biodiversity discussed above, can we expect similar consequences (such as the possibility of extinction of pure parental genomes) in the case of a rare taxon such as *J. erucifolia* in Poland? We will attempt at least a preliminary assessment of these threats. We hope to get a more complete answer once the ongoing studies on other *J. erucifolia* populations are completed.

## 2 MATERIALS AND METHODS

### 2.1 Plant material

Fresh plant material for molecular studies was collected in natural populations from 19 specimens of *J. vulgaris*, 20 specimens of *J. erucifolia*, and 25 putative interspecific hybrids of *J. vulgaris × J. erucifolia*. All putative hybrids were collected from the two localities: 15 individuals from Mięćmierz (Mm1–Mm15) and 10 individuals from Pęczelice (Pc1–Pc10). Material of both supposed parental species came from various regions of Poland. Specimens of *J. vulgaris* were collected in Golęcin (10 individuals, G1–G10) and the remaining samples (nine individuals, Pu1–Pu9) near Puławy, relatively close to the occurrence of hybrids. Because of the genetic variation within populations of *Jacobaea* species, at least in the case of *J. erucifolia* seems to be higher than between populations (B. Gawrońska, M. Marszałek, unpublished data, research in progress), four individuals were taken from each of the four presently existing *J. erucifolia* subsp. *erucifolia* populations in the Western Pomerania. In addition, four samples were collected from individuals previously described as *J. erucifolia* subsp. *tenuifolia*, in the region of origin of the hybrids (near Pęczelice); thus, a greater number of different populations will allow for covering most of the intraspecific variation. For details concerning examined specimens, see Table 1.

**TABLE 1:** Origin of sampled populations of the studied species.

Population	Acronym	Latitude N	Longitude E	Altitude (m a.s.l.)
*J. erucifolia* subsp. *erucifolia*				
Szczecin Skolwin	S	53°30′	14°36′	58
Szczecin Stołczyn	St	14°36′	53°30′	3
Moczyły	M	53°19′	14°28′	3
Kłosów	K	52°44′	14°27′	35
*J*. *erucifolia* subsp. *tenuifolia*				
Pęczelice	P	50°26′	20°47′	242
*J. vulgaris*				
Poznań (Golęcin)	G	52°25′	16°51′	83
Puławy	Pu	51°24′	21°58′	115
Putative hybrids				
Męćmierz	Mm	51°18′	21°54′	150
Pęczelice	Pc	50°26′	20°47′	242

### 2.2 DNA extraction

Young leaves collected in the field were dried with silica gel. The total genomic DNA of all 64 specimens was extracted following the method described by Doyle and Doyle (1987). The contaminating RNA was removed by digestion with RNase A. DNA quality and concentration were estimated by electrophoresis and spectrophotometry, adjusted to 20 ng/μL, and used as a template in polymerase chain reaction (PCR).

### 2.3 AFLP procedure

Sixty‐four individuals representing two taxa and putative hybrids were included in the AFLP analysis. All sequences of primers and AFLP adapters were based on Vos et al. (1995). Detailed methods for the preparation and program of PCR reactions were provided by Gawrońska et al. (2019). Four individuals of pure *J. vulgaris* and pure *J. erucifolia* were initially tested with 15 different selective primer combinations. From this group, primer pairs producing bands that seemed to distinguish the two species were selected, and these primers were further screened over all individuals. For selective amplification, seven *Eco*RI/*Mse*I (+3/+3) primer pairs (E‐AAG/M‐CTG, E‐ACA/M‐CAG, E‐ACA/M‐CAT, E‐ACA/M‐CCG, E‐AGG/M‐CAG, E‐AGG/M‐CTG, and E‐AAG/M‐CTG) were tested to find specific diagnostic markers for each parental species. Each reaction was repeated at least two times (repeat runs were made to check for reproducibility). Amplification products were separated and detected on an ABI Prism 3500 capillary sequencer. GeneScan 600 LIZ‐labeled size standard (Applied Biosystems) was used for fragment sizing. The fluorescent AFLP patterns were evaluated using GeneMarker version 1.6 (Softgenetics LCC). Fragments ranging from 50 to 500 bp were automatically scored as present (1) or absent (0) and manually corrected after visual inspection of the electrophoretograms.

### 2.4 ITS sequence analysis

The entire ITS region (ITS1, 5.8S, ITS2) was amplified using the universal ITS1 and ITS4 primers developed by White et al. (1990). Reaction conditions were described previously by Gawrońska et al. (2019). Efficiency and quality of amplification was verified by the electrophoretic separation of 5 μL of PCR products in 1.5% agarose gel. Double bands of similar length, difficult to separate from each other, were observed in some of the analyzed PCR products. It is known that direct sequencing in such cases usually produces chimeric or unreadable peaks in the chromatogram; therefore, cloning and subsequent sequencing are necessary. As the aim of the analysis was to detect copies of both parental species in the hybrids (preferably without the need for subcloning PCR products and sequencing of different clones), the double products were separated in a polyacrylamide gel. After silver staining, individual bands were excised from the gel and DNA was recovered, purified, and re‐amplified. All PCR products were sequenced in both directions using the same primers as during amplification. The 15 different ITS sequences generated in this study were deposited in GenBank (accession numbers: OP764514–OP764528).

### 2.5 Chloroplast DNA sequence analysis

As the mode of inheritance of cpDNA in Senecioneae, according to Harris and Ingram (1992) is strictly maternal, examination of relationships with cytoplasmic markers allows the identification of maternal and paternal parents. In the present study, an intergenic spacer between the *trn*L (UAA) 3′ exon and *trn*F (GAA) was amplified using universal primers (e, f) described in Taberlet et al. (1991). PCR reactions were carried out in a total volume of 25 μL containing 20 pmol each of primer and 40 ng of template DNA using the KAPA3G Plant PCR Kit (Sigma Aldrich). Reactions were performed in a PTC‐100 thermal cycler (MJ Research), programmed with preliminary denaturation of DNA for 5 min at 95°C, 30 cycles of denaturation for 45 s at 92°C, primer annealing for 45 s at 53°C and for 2 min at 72°C followed by 1 cycle for 10 min at 72°C. The amplification efficiency was verified by the electrophoretic separation of 5 μL of PCR product in 1.5% agarose gel. PCR products were purified using the kit for cleanup DNA after enzymatic reaction (Clean‐Up, A&A Biotechnology Company) and sequenced in both directions using the same primers as in the amplification. Then, sequences of putative hybrid *J. vulgaris* × *J. erucifolia* were compared with those of its respective parents. The four different cpDNA sequences generated in this study have been deposited in GenBank (accession numbers: OP806269–OP806272).

### 2.6 Data analysis

The distribution of bands across taxa as well as the number of polymorphic bands, the average number of bands per primer, and bands shared among species were examined. Apart from monomorphic (bands present in all specimens under the study), polymorphic (bands present in at least one but not all specimens), group of unique bands which were present in at least one individual in a given taxon and not present in any other were also defined (Delaporte et al., 2001). However, for the identification of hybrids, finding diagnostic markers is of paramount importance. Those diagnostic markers (a subset of all unique ones, always present in one species and not present in another) are known as species‐specific or private. Analyses of molecular variance (AMOVA; Excoffier et al., 1992) were carried out in GenAlEx 6.1 (Peakall & Smouse, 2007).

The binary matrix of AFLP phenotypes was the basis for calculating Dice similarity and distance matrices. The distance matrix was then used to construct the unrooted neighbor‐joining (NJ) dendrogram and phylogenetic network based on the Neighbor‐Net algorithm using the SplitsTree4 v.4.13.1 software (Huson & Bryant, 2006). Neighbor‐Net (Bryant & Moulton, 2004) visualizes better hybridization and reticular relationships among individuals. The analyses mentioned above were supplemented with the unweighted pair group method with arithmetic mean (UPGMA) dendrogram and principal coordinate analysis (PCoA), both performed in PAST 3.15 (Hammer et al., 2001). Bootstrapping was used to calculate the support value for each node on dendrograms (1000 repeats). To identify genetic groups without a priori knowledge of sample origin, we used the model‐based Bayesian approach implemented in STRUCTURE, version 2.3.4 (Pritchard et al., 2000). STRUCTURE analysis was performed based on the admixture model, with the recessive allele option set to 1 as AFLPs are the dominant markers (Falush et al., 2007). Ten independent repetitions were performed for each number of groups (K) ranging from one to five, with 10^5^ steps burn‐in followed by 2 × 10^5^ MCMC iterations. The consistency of the results in 10 replicates of analyses was assessed using CLUMPAK (Kopelman et al., 2015), and the best number of clusters was determined by the Evanno ΔK method (Evanno et al., 2005) implemented in the online STRUCTURE HARVESTER program (Earl & von Holdt, 2012). The INTROGRESS package (Gompert & Buerkle, 2010), implemented in the R software environment (R Core Team, 2018), was used to calculate the hybrid indices for each individual based on the AFLP binary matrix; putatively pure *J. vulgaris* and *J. erucifolia* specimens, based on their morphology, were selected as parental groups.

The results of the analyses described above are graphically presented using a uniform color code system: red—*J. vulgaris*, blue—*J. erucifolia*, and green—putative hybrids.

### 2.7 Sequence data

All DNA sequences (ITS and cpDNA) were visualized and analyzed using SnapGene Viewer 6.0.2. (https://www.snapgene.com/snapgene‐viewer), adjusted visually where needed and automatically aligned using the program MEGA 11 (Tamura et al., 2021). To elevate the discrimination power of analyses, sequences of both species available in GenBank were also downloaded and compared with those obtained in the present work.

## 3 RESULTS

### 3.1 AFLP analyses

Seven of the 15 initial AFLP primer combinations produced clear and reproducible fragments and yielded a total of 920 amplification products sized from 50 to 500 bp, all were polymorphic across all 64 accessions. The number of polymorphic bands per primer combination ranged from 114 (*Eco*ACA/*Mse*CAT) to 142 (*Eco*AGG/*Mse*CAG) with an average of 131.4 bands. However, in particular groups of species (*J. vulgaris*, *J. erucifolia*, and putative hybrids), the percentage of polymorphic bands was variable and dependent on the primer combination used. Generally, populations of *J. vulgaris* showed lower genetic variation than that of *J. erucifolia* and hybrids (polymorphism was 94.5%, 99.5%, and 99.6%, respectively; for details see Table 2). The AMOVA showed that the main part of the total genetic differentiation (77%) was located within populations, while 12% of the variation was among populations. Moreover, 154 (16.74%) of the total polymorphic bands distinguished the two parents. The two taxa, *J. vulgaris* and *J. erucifolia*, had 73 and 81 unique bands, respectively, and both sets of them were observed in putative hybrids. Some of these unique bands have been classified as diagnostic (species‐specific) for each parental species. Fourteen of the 32 such bands detected in *J. vulgaris* were fully diagnostic (present in 100% of the plants and completely absent in *J. erucifolia*), while eight others were found in most *J. vulgaris* individuals. In the *J. erucifolia* group, 24 diagnostic bands were detected. Only two of them were present in the profiles of almost all accessions. Most of the remaining 22 bands showed significant differences in the frequency of occurrence across individual profiles. Almost all of the DNA bands amplified from the putative hybrids co‐occurred in either *J. vulgaris* or *J. erucifolia* banding patterns. Furthermore, some bands unique for putative hybrids also were observed.

**TABLE 2:** Seven selected primer combinations, number of AFLP loci, and degree of polymorphism (%) in total as well as in populations of *Jacobaea vulgaris* (19 accessions), *Jacobaea erucifolia* (20 accessions) and their putative hybrids (25 accessions).

Primer combination	Total products	*J. vulgaris*			*J. erucifolia*			Putative hybrids		
		Products	Monomorphic bands	Polymorphism (%)	Products	Monomorphic bands	Polymorphism (%)	Products	Monomorphic bands	Polymorphism (%)
Eco ACA/Mse CAT	114	81	2	97.5	93	0	100	106	0	100
Eco AGG/Mse CTT	140	123	1	99.2	123	0	100	130	2	99.5
Eco ACA/Mse CAG	134	103	28	72.2	121	0	100	128	0	100
Eco ACA/Mse CCG	137	110	8	92.7	126	0	100	134	0	100
Eco AGG/Mse CAG	142	127	1	99.2	134	1	99.3	128	1	99.2
Eco AAG/Mse CTG	123	105	1	99.0	112	1	99.1	106	0	100
Eco ATC/Mse CTG	130	115	1	99.1	122	1	99.4	122	0	100
Total	920	764	42	94.5	831	4	99.5	854	3	99.7

**FIGURE 1:**
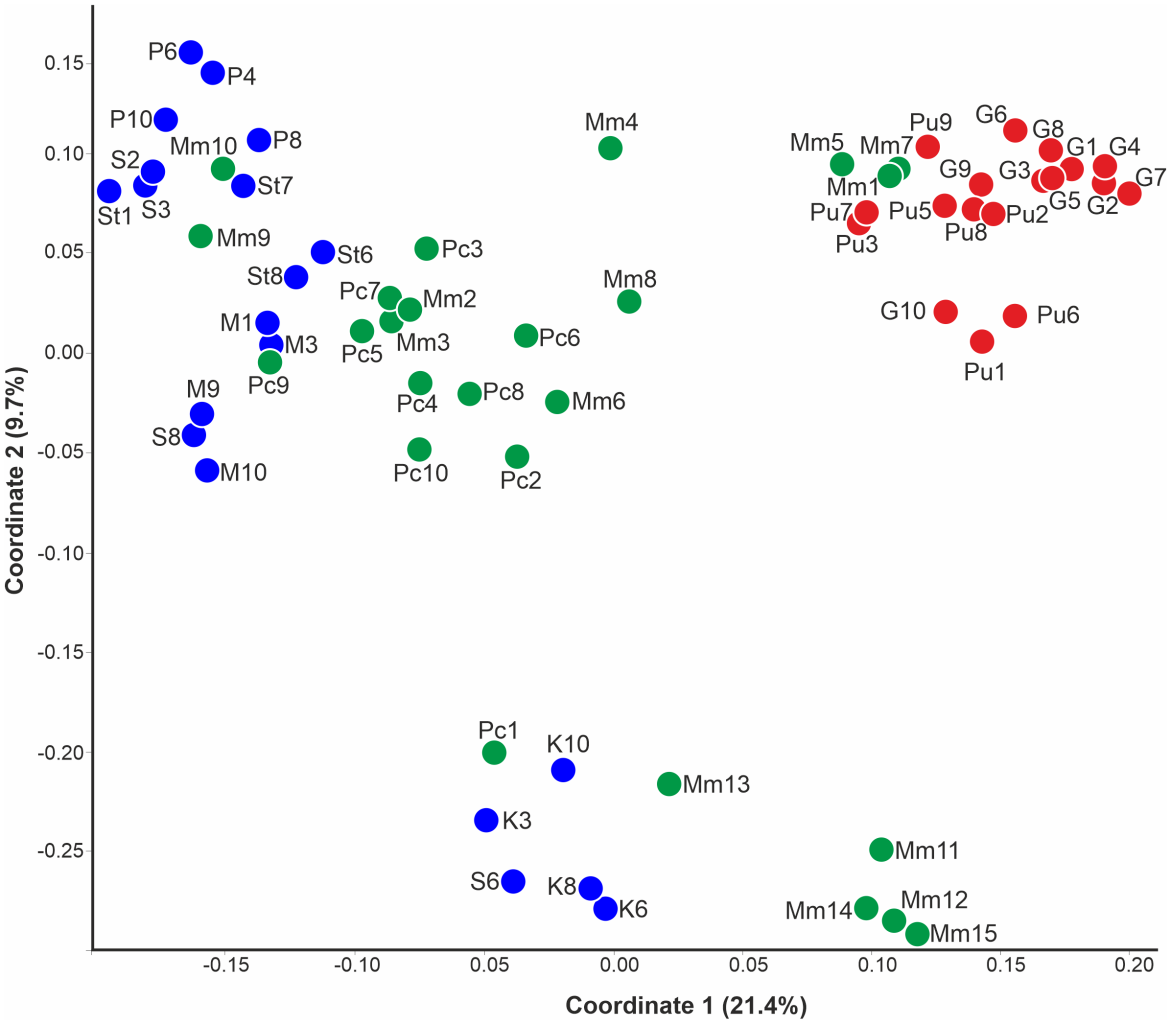
Principal coordinates analysis (PCoA) plot based on the individual genetic distance calculated with 920 AFLP markers. The first two axes explain 26.2% and 9.7% of the variation, respectively. Individuals are represented by dots in colors, (red corresponds to *Jacobaea vulgaris* dark blue corresponds to *Jacobaea erucifolia*, and green corresponds to hybrids).

**FIGURE 2:**
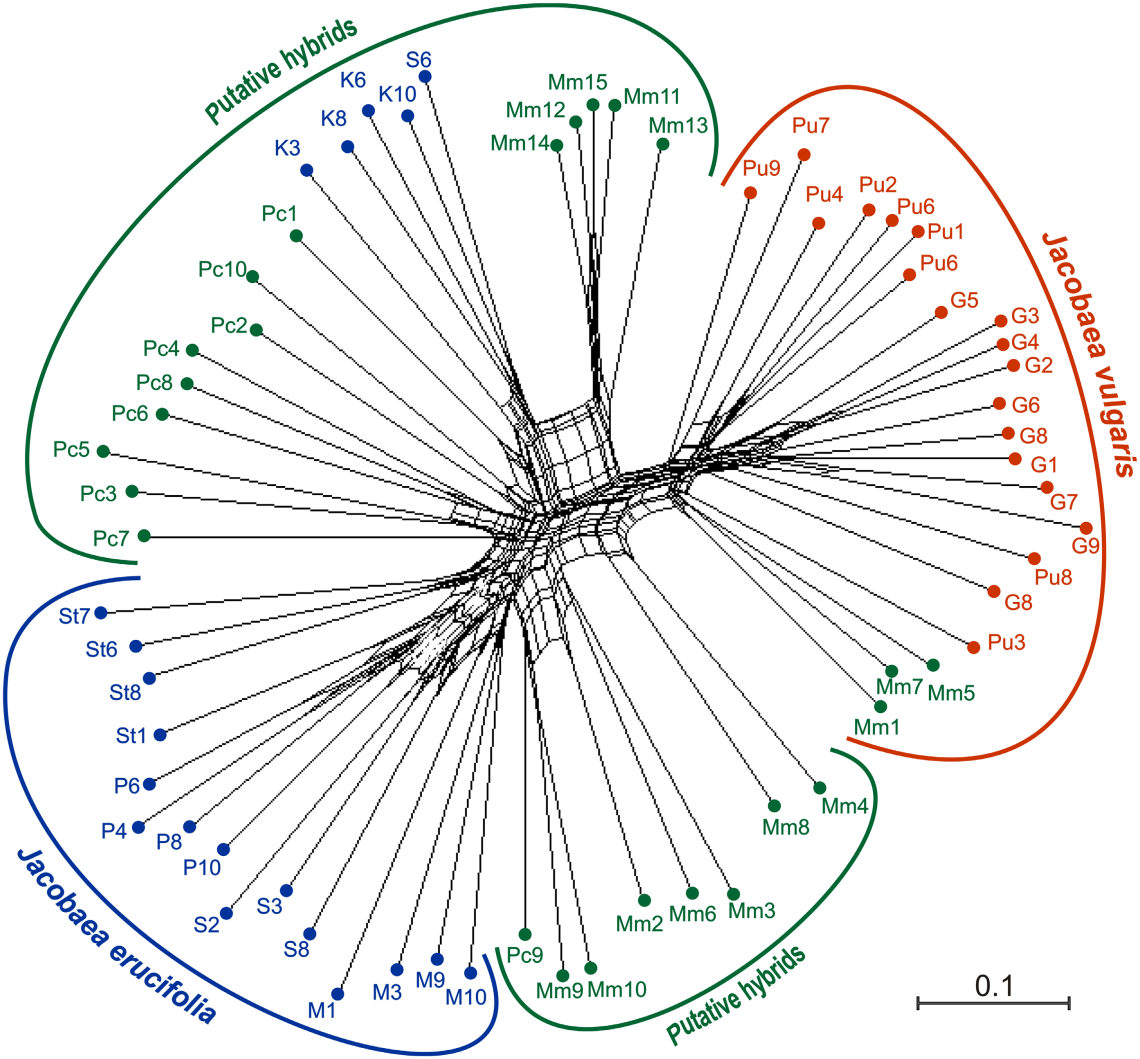
Neighbor‐Net derived from AFLP binary matrices of 64 individuals representing *Jacobaea vulgaris*, *Jacobaea erucifolia*, and their putative hybrids. It should be noted that putative hybrids form clusters corresponding to the individual sites of their occurrence (Mięćmierz and Pęczelice).

Cluster analyses were performed based on data from polymorphic AFLP bands (combination of 920 markers). PCoA (Figure 1) divided all accessions into three groups between the two first coordinates, which accounted for 31.1% of the total genetic variation (21.4% and 9.7% for the first and second axes, respectively). Three distinctly isolated groups encompassed individuals identified by morphological characteristics as parental species and putative hybrids. Most of the putative hybrids (Pc2‐10, Mm2‐4, Mm6, and Mm8‐10) formed a loose group with *J. erucifolia*, however, showing some distinctiveness. The separate group consisted of accessions previously defined as pure *J. erucifolia* (population K and individual S6) and putative hybrids (Mm11‐15, Pc1) and was located between the group of *J. erucifolia* and the group encompassing samples of *J. vulgaris* and three putative hybrids (Mm1, 5, 7). The Neighbor‐Net network (Figure 2) also confirmed, to an even greater extent, the separation of the two parental taxa and intermediate positioning of putative hybrids which formed more defined subgroups. Except for three (Mm1, 5, and 7), all putative hybrids formed several smaller clusters between the main groups of parental species. Most of the hybrids (17 out of 25) as well as a few individuals of *J. erucifolia* formed two clades close to *J. erucifolia*, indicating that they were likely products of backcrossing to *J. erucifolia*. The Mm11–Mm15 hybrids formed a separate clade. The parental individuals of *J. erucifolia* (populations M, P, S, St) formed one cluster, while *J. vulgaris* individuals and the remaining tree hybrids (Mm1, 5, and 7) clustered in other. The analysis also showed that individuals of all surveyed species collected in particular localities clustered together and may reflect some genetic distinction related to the geographic isolation of populations (Figure 2).

**FIGURE 3:**
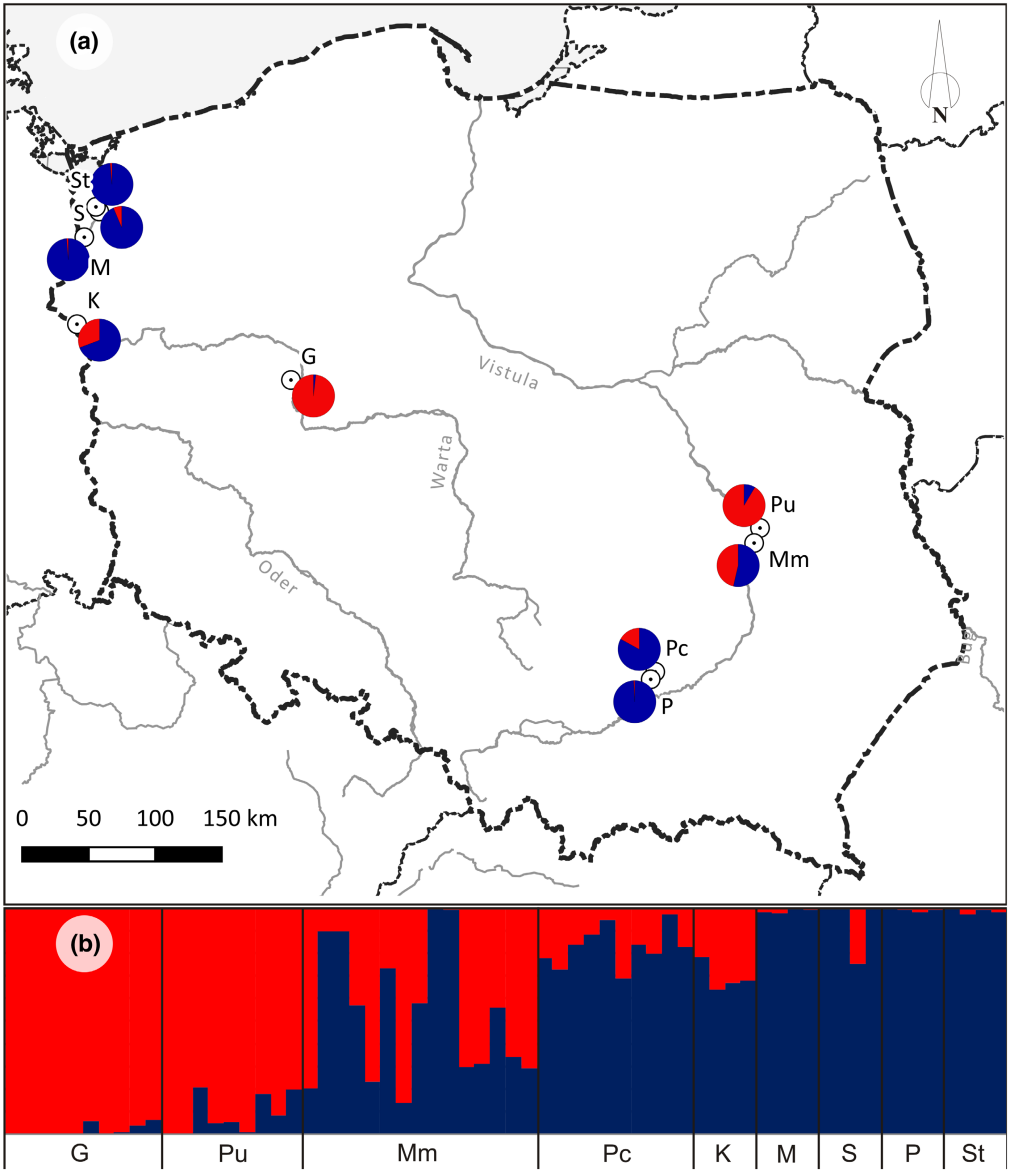
Clustering analysis of AFLP data for a pure *Jacobaea vulgaris* population, pure *Jacobaea erucifolia* population, and hybrid population and their distribution in Poland. (a) A map of Poland showing the location of individual surveyed populations (parental species and putative hybrids). (b) Clustering analysis using STRUCTURE. Individuals are represented by columns, with colors (red corresponds to *J. vulgaris* and dark blue corresponds to *J. erucifolia*) representing the proportion of their genome assigned to the inferred *K* = 2 clusters in the model‐based admixture analysis. Symbols identify the different populations investigated. Additional details for each population are given in Table 1.

In the STRUCTURE analysis, including all populations, the best‐supported number of genetic clusters was found to be two (*K* = 2). The detected differentiation pattern agreed with the sample partition into three groups. Two were represented by tentatively parental, pure species populations (except for population K). They were quite homogenous and consisted of individuals assigned nearly entirely to a single cluster (*J. vulgaris* populations G and Pu to Cluster 1; *J. erucifolia* populations M, P, S, and St to Cluster 2). The third group, encompassing populations Mm and Pc (morphologically defined as a hybrid) and population K (assigned provisionally to *J. erucifolia*), showed a distinct overlap of the gene pools of the two previous groups. In the latter two populations, the cluster typical of *J. erucifolia* dominated clearly. The Mm population was the most diverse because it included specimens with the pattern of pure *J. erucifolia* (or with the domination of its gene pool), and those in which the cluster present in *J. vulgaris* predominated. On the other hand, one sample of “pure‐species” population S (*J. erucifolia*) seemingly combined genetic material from the other parental species (similarly to all samples from population K). A slight admixture of the *J. erucifolia* gene pool was also present in several individuals of *J. vulgaris* population Pu (Figure 3b).

**FIGURE 4:**
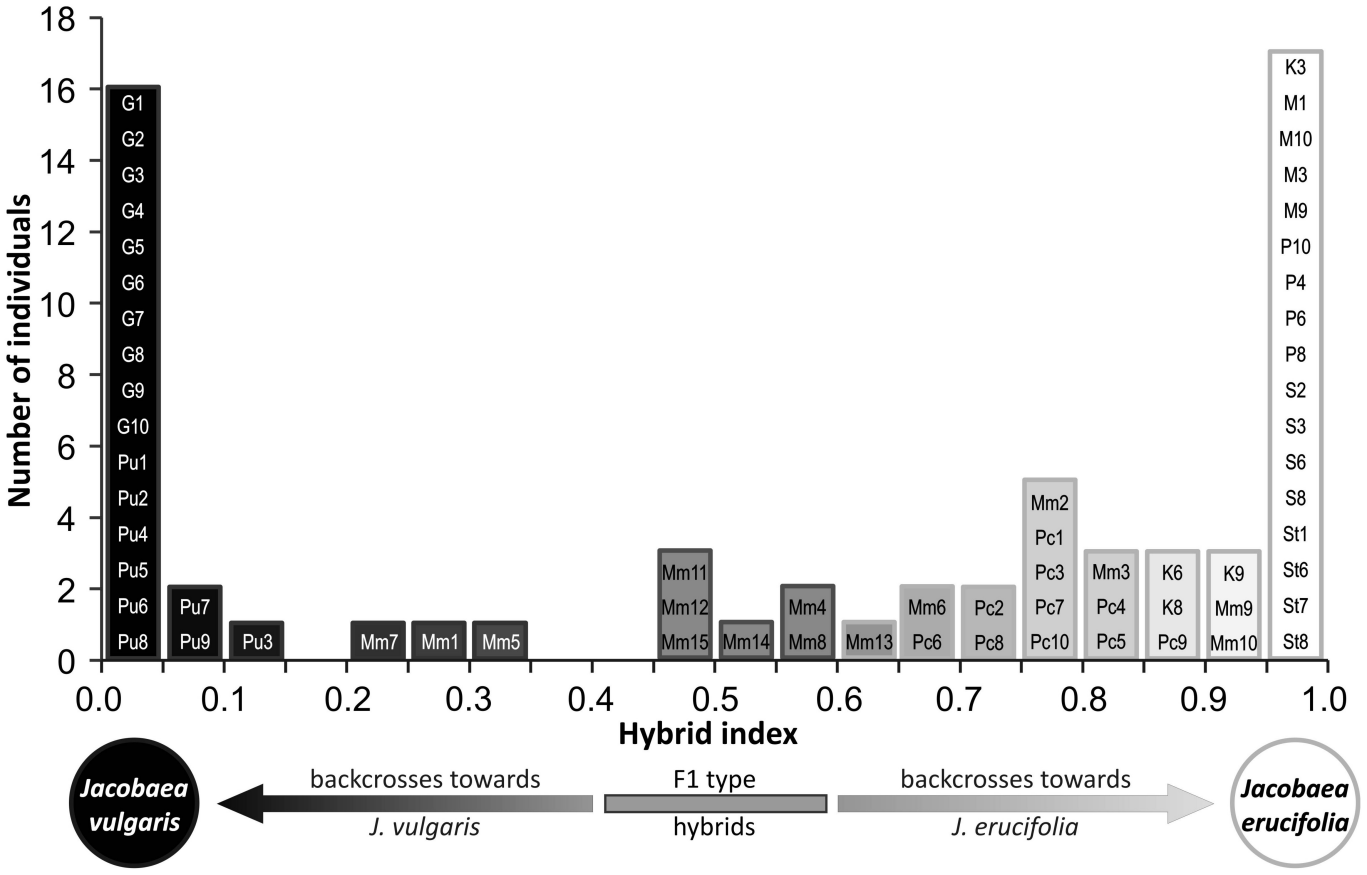
Frequency distribution of individuals versus the hybrid index derived from the AFLP data. The figure shows groups of the pure or nearly pure parents (index = 0 or 1), a cluster of probable F1 hybrids (index = 0.466–0.580), and probable backcrosses to *Jacobaea vulgaris* (index = 0.233–0.309). Other intermediate plants may be complex backcrosses, most individuals are similar to one of the parental taxa, *Jacobaea erucifolia*.

Finally, hybrid indices were calculated using the same data set. AFLP analysis confirmed the field identification of 16 plants as pure *J. vulgaris* (index = 0). Another three plants (0.061–0.119) represented nearly pure parental plants. As in the above case, 17 specimens identified as *J. erucifolia* (index = 1 or index = 0.966–0.986) were classified as pure. Six others (index = 0.855–0.948) comprising individuals of *J. erucifolia* (population K) and three plants, tentatively identified as hybrids, were also interpreted as nearly pure. Among the putative hybrids, none exhibited perfect marker additivity as expected in F1 (index = 0.5); however, several of them had an index very close to this value (e.g., Mm12 and Mm14 had an index = 0.496 and 0.513, respectively) and were therefore interpreted as F1‐type. Three putative hybrids (index = 0.234–0.309) were likely to be backcrosses toward *J. vulgaris* while the other 12 (index = 0.649–0.841) were diagnosed as probable backcrosses towards *J. erucifolia* (Figure 4).

### 3.2 Analysis of nuclear ribosomal DNA

Forty ITS sequences from the putative hybrids (some had two ITS sequence variants) and the parental species were aligned and compared. Sequence analysis of several parent specimens, both in the *J. vulgaris* and *J. erucifolia* group, did not show any differences in the sequence. Furthermore, except for three substitutions (positions 118, 390, and 607), sequences of *J. vulgaris* obtained in this study, and that deposited in GenBank (accession number: GU818567) were also identical. This within‐individual polymorphism may indicate that *J. vulgaris* contains two separate ITS copies that differ by three substitutions. Position 607 was also polymorphic in the sequences of most hybrids.

**FIGURE 5:**
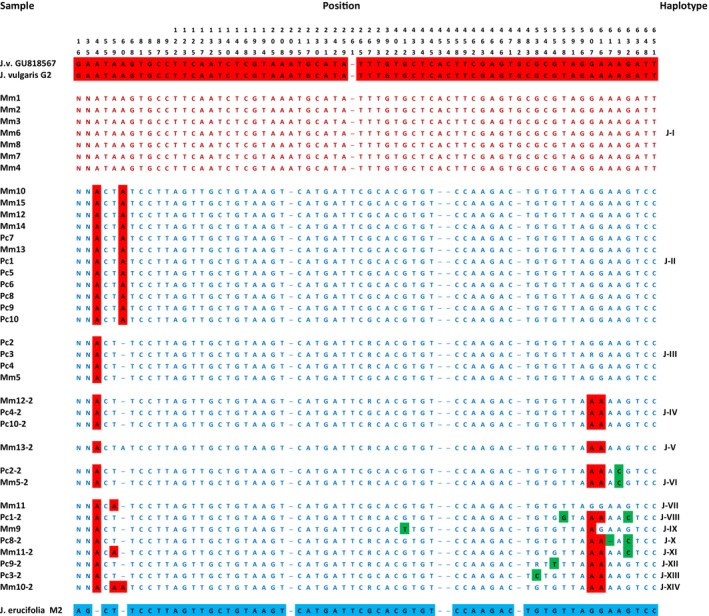
Variable nucleotide sites in internal transcribed spacer sequences of *Jacobaea vulgaris*, *Jacobaea erucifolia*, and their putative hybrids (Mm and Pc). Numbers in accession names, before “‐” are population sample numbers, while number 2, after “‐” is a variant of the ITS sequence of each accession. The positions of the variable nucleotide sites are relative to the first sample. Nucleotide ambiguity cod R = G/A; dashes indicate deletion, N = unavailable.

The total length of ITS sequences varied from 685 to 691 bp. The parental sequences differed in 58 variation sites, including 52 substitutions and six indels (insertion/deletion). As a result of comparing the ITS sequences of hybrids, 66 variation sites, including 59 substitutions and seven indels, were found. These 66 variation sites divided the ITS sequences of hybrids into 14 distinct haplotypes (defined here as haplotypes J‐I–J‐XIV). Seven of the sequences derived from the putative hybrids were identical to *J. vulgaris* (100% match; haplotype J‐I). The remaining sequences showed significant sequence similarity to *J. erucifolia*. Nineteen of these sequences were almost identical to *J. erucifolia*: haplotypes J‐II (97% match), J‐III (98.5% match), and J‐IV (95.5% match), found in 12, 4, and 3 accessions, respectively. Haplotype II differs from the other two mainly by the lack of a 1‐bp indel (position 60). Other haplotypes were found in two: J‐VI (93.9% match) or in only one accession (J‐V and J‐VII–J‐XIV). Apart from the J‐VII haplotype (97% match), in others, the sequence similarity to *J. erucifolia* was in the range of 92.4%–93.9%. Their sequences were similar to each other and the differences concerned only a few single nucleotides. The additive sites of two putative parental species were detected in the hybrid sequences proving that all putative hybrids were indeed hybrid derivatives of *J. vulgaris* and *J. erucifolia*. In addition, within the sequences of some hybrids, the presence of single nucleotides and one deletion, absent in the sequences of any of the parent taxa, was also observed. These data are summarized in Figure 5.

### 3.3 Analysis of chloroplast DNA

The aligned sequences of the chloroplast intergenic spacer between *trnL* (UAA) 3′ exon and *trn*F (GAA) were 396 bp in length. Sequences of 25 putative hybrids, one individual of *J. vulgaris* (sequences of several analyzed individuals were identical) and four individuals from populations of *J. erucifolia* were compared. We took into account previous results, showing that small admixture of the *J. vulgaris* gene pool was also present in individuals of “pure‐species” populations of *J. erucifolia* (S and K). Two individuals from each of these groups were selected for sequencing, while next two were randomly selected.

Parental sequences comparison showed that they differed by three nucleotide substitutions (positions: 45, 87, and 198) and one 10‐bp indel (positions 268–277). Twenty‐three of the 25 morphologically intermediate individuals exhibited chromatogram additivity on these differentially fixed sites between *J. vulgaris* and *J. erucifolia*. All, except one different nucleotide (position 45), had the same sequence as *J. vulgaris* (haplotype H1). The remaining two individuals (Mm9 and Mm10) had an identical sequence with *J. erucifolia* on these four fixed sites (haplotype H2). Moreover, within individuals of *J. erucifolia*, two genotypes divided by one variation (position 45) were detected. S1, P1, and P10 individuals had a G nucleotide, while K3 individual had a T nucleotide at this position (haplotype H3; Table 3). These results suggest that both parents served as the mothers of the hybrid progeny. It also indicates that hybridization is possible in both directions, but *J. vulgaris* was the most frequent maternal parent.

**TABLE 3:** Nucleotide positions in the aligned *trn*L‐F intergenic spacer sequences that differ between *Jacobaea vulgaris*, *Jacobaea erucifolia*, and their putative hybrids.

Taxon			Variable sites			
			45	87	198	230–277
*J. vulgaris*	Pu3		T	C	G	GCATGAGACT
Hybrid	Mm1‐8, 11‐15, Pc1‐10	H1 (23)	G	C	G	GCATGAGACT
Hybrid	Mm9‐10	H2 (2)	G	A	C	‐‐‐‐‐‐‐‐‐‐‐‐‐‐‐‐‐‐
*J. erucifolia*	K3	H3 (1)	T	A	C	‐‐‐‐‐‐‐‐‐‐‐‐‐‐‐‐‐‐
*J. erucifolia*	S1, P1, P10		G	A	C	‐‐‐‐‐‐‐‐‐‐‐‐‐‐‐‐‐‐

*Note*: Three haplotypes are found, 23 and 2 hybrid individuals have haplotype H1 and H2, respectively, while one accession from the population K (*J. erucifolia*) has haplotype H3.

## 4 DISCUSSION

Hybridization is generally considered a natural phenomenon in both the plant and animal worlds. Morphological intermediates are usually suggested to be derived from direct hybridization or introgressive hybridization of the two distinguished species. However, many examples show that distinguishing hybrids is not so easy, especially if no single morphological feature allows you to clearly distinguish the analyzed plants. The definition of the boundaries between “typical” and ‘intermediate’ units is often more or less arbitrary (Aas, 1993). Moreover, morphologically intermediate forms that are suspected of being hybrids are regularly observed in natural mixed populations. In addition, a high degree of morphological variation in plants can often be explained by phenotypic plasticity (Le Roux et al., 2006).

Hybridization within Senecioneae species is well known. They hybridize with other congeners when they co‐occur. As mentioned above (introduction), hybrids between *J. vulgaris* and *J. erucifolia* were recorded but not confirmed by any research. Moreover, Benoit et al. (1975) challenged the reliability of the United Kingdom and Continental records of hybrids between *J. vulgaris* and *J. erucifolia*.

The above information and the analysis of the characteristics of both species indicate that interspecific hybridization between *J. vulgaris* and *J. erucifolia* is possible. Both species are closely related according to their phylogeny and morphology (Pelser et al., 2002, 2003, 2004). According to Qiu et al. (2011), the likelihood of hybridization between two closely related species is usually very high. They are both tetraploids and occupy similar habitats, massively spreading into various anthropogenically disturbed habitats such as roadsides, pastures, abandoned vineyards, and waste areas (Hodalova et al., 2015; Leiss & Müller‐Schärer, 2001). Furthermore, following some authors (Anderson, 1948; Anderson & Stebbins, 1954; Leiss & Müller‐Schärer, 2001), habitat disturbance generates novel ecological niches between hybridizing species that allow diverse forms such as stabilized introgressants and homoploid hybrid species to persist. Such examples have also been described in Senecioneae (Abbott et al., 2003; Brennan et al., 2012; Lowe & Abbott, 2015). Another factor that might contribute to the natural hybridization between *J. vulgaris* and *J. erucifolia* is the long and partially overlapping flowering periods. The average flowering time of *J. vulgaris* is generally between June and August, while *J. erucifolia* flowers between July and September. However, isolation by flowering time is incomplete and hybridization is possible in such cases (Brennan et al., 2009; Chapman et al., 2005). Moreover, they may share the same or similar pollinators, for example, 36 insect species (mainly Diptera) pollinate *J. vulgaris* (Jung et al., 2020). It is very likely that at least some of them also pollinate *J. erucifolia* which may greatly increase the possibility of hybridization.

Since 25 specimens showed morphological traits intermediate between *J. vulgaris* and *J. erucifolia*, we hypothesized that they might be hybrids. The results of studies using AFLP seem to fully confirm this hypothesis. This method is commonly known as generating a unique fingerprint of a specific genome. Compared to other analyses, it generates a higher number of products (polymorphic fragments and unique bands) in one reaction and is highly reproducible.

A high and comparable level of polymorphism was detected among the 64 genotypes representing two *Jacobaea* species and their putative hybrids. An AMOVA revealed that the main part of AFLP variation was due to differences between individuals within populations (77%), while 12% accounted for the inter‐population component. Similar results for *J. vulgaris* and *J. aquatica* populations have been reported previously (Abbott et al., 2009; Hodalova et al., 2015; Jung et al., 2020; Winter et al., 2013). Of the total amplified polymorphic products, 154 (16.74%) distinguished the two parental species (part of these bands were diagnostic for each species). In addition, the presence of unique bands, absent in the profiles of the parents, was observed in the profiles of the hybrids. Such hybrid‐specific bands (not seen in parental genomes), according to some authors, can be generated as a result of recombination and mutation in meiosis during hybridization (Darnell et al., 1990; Huckett & Botha, 1995) or may also be created by heteroduplex formation (Ayliffe et al., 1994). However, it is unclear whether these novel bands may be responsible for new genes associated with important traits (Zhao et al., 2017).

All analyses based on the AFLP data clearly distinguished *J. vulgaris* from *J. erucifolia* and confirmed the separate and intermediate status of the individuals classified as putative hybrids. Most of the putative hybrids grouped with *J. erucifolia*, but always as distinct subclades, indicating that they were likely products of backcrossing to *J. erucifolia*. Only three putative hybrids (Mm1, 5, and 7) were in the clade of *J. vulgaris*.

Bayesian analyses using STRUCTURE clustered the accessions similarly confirmed by previous results (NJ, PCoA) and showed the admixture of parental gene pools in several individuals tentatively classified as “pure species” based on morphology. Population clustering demonstrated a consistent pattern, including independent clusters formed by individuals from pure populations with hybrids exhibiting an admixed genotype. Moreover, a distinct overlap of the gene pools of the parental groups was also observed in all analyzed specimens from population K and one from population S, which were preliminary assigned to “pure‐species” *J. erucifolia*. A slight admixture of the *J. erucifolia* gene pool was also present in several individuals of the *J. vulgaris* population Pu. This might indicate the repeated backcrossing of an interspecific hybrid with *J. vulgaris*. Both parental gene pools contributed to the genotypes in the hybrid population, but *J. erucifolia* generally had a more considerable share in them. Nevertheless, the highly diverse hybrid population Mm also included specimens in which the cluster present in *J. vulgaris* predominated, as well as those with the pattern of nearly pure *J. erucifolia*.

Finally, AFLP provided a measure of the nuclear composition of 30 putative hybrids (25 intermediate morphological individuals and those indicated as putative hybrid origin by STRUCTURE analysis). It showed that 20% (six individuals) of all putative hybrids might be classified as F1‐type with a hybrid value of 0.466–0.580. The remaining 24 plants in the hybrid group had lower or higher hybrid indices. Such values suggest that they are rather later‐generation backcrosses containing a high proportion of the *J. erucifolia* or the *J. vulgaris* genome. On the other hand, more than half of all individuals (63.3%) had a hybrid index larger than 0.6, which confirms the results of other analyses, indicating that most individuals had a more significant fraction of *J. erucifolia* in their genome.

Summing up, the results of the Bayesian admixture analyses using STRUCTURE indicated that introgression was asymmetric, with *J. erucifolia* contributing more to the genomic composition of the hybrids than *J. vulgaris*. Such hybrids demonstrated a closer relationship to one parent, usually suggesting frequent backcrossing (Clarkson et al., 2011; Winter et al., 2013). Furthermore, hybrid indices showed that backcrosses with *J. vulgaris* were rare (three individuals).

Some authors (Hegarty & Hiscock, 2005; Schwarzbach & Rieseberg, 2002) point to the importance of not relying on a single‐marker system when studying complex genetic events like speciation/hybridization. In their view, such studies require accumulating of evidence from multiple sources before definitive answers can be given. This is why in addition to the AFLP method, biparentally inherited nuclear markers and maternally inherited plastid markers were also applied in this study. Sequence data further showed that the ITS sequences from *J. vulgaris* and *J. erucifolia* differed significantly. Both haplotypes were found in most putative hybrids with several substitution or indel differences (haplotypes J‐I, J‐II, and III, respectively). Such minor differences in ITS sequences in the remaining haplotypes were also observed. Again, evidence from both the ITS sequence comparison and the ITS phylogenetic tree (not shown) supported the significant similarity of most hybrids to *J. erucifolia*. At the same time, additive sites of two putative parental species were also detected, providing evidence that all putative hybrids were indeed hybrid derivatives. In addition, single substitutions and one deletion were observed in the hybrid sequences, which were not found in the sequences of either parent species. As with bands found only in AFLP profiles of some hybrids, interpretation is difficult. Perhaps we are dealing with earlier involvement of another species or ancient hybridization, but confirmation of either hypothesis requires research covering larger areas of the genome.

Each morphologically intermediate individual and putative hybrid K3 had a chlorotype identical to either *J. vulgaris* (23 accessions) or *J. erucifolia* (three accessions). It indicated that the hybridization was asymmetrical and bidirectional because chloroplast DNA is maternally inherited in most angiosperms, including *Senecio*‐*Jacobaea* complex (Harris & Ingram, 1992).

The results of previous analyses based on AFLP data, combined with the sequence analysis, provide compelling evidence for the natural hybridization between *J. vulgaris* and *J. erucifolia*. The status of two hybrids (Mm9 and Mm10) may raise some doubts. They clustered in Nj and PCoA with other hybrids, while having *J. erucifolia* chlorotype and being pure or almost pure *J. erucifolia* according to STRUCTURE and hybrid index analysis. At the same time, they had additive sites in the ITS sequence and diagnostic markers of the other parent. The presence of unique markers from both parents is believed to identify the presence of a hybrid (Kirk, Choi, et al., 2005; Kirk, Vrieling, & Klinkhamer, 2005). Following this definition, the question arises whether in such cases the difficulty in confirming hybrid origin may be due to the insufficient sensitivity of the methods. According to Fritz et al. (1994), increasing the number of markers in the analysis will make the exact genetic classification of hybrid plants more reliable. Boecklen and Howard (1997) suggest that more than 70 diagnostic markers are needed to distinguish parent species from advanced backcrosses with sufficient certainty. On the other hand, highly backcrossed hybrids can be difficult to distinguish from parental individuals because such individuals often cluster with pure individuals.

Our analyses unquestionably confirmed the presence of hybrids. However, the unanswered question, at least for now, is whether the process of natural hybridization is just a result of co‐occurrence and taxonomic proximity, or whether the reasons are much more complex and should raise legitimate biodiversity concerns. Both taxa occupy ruderal habitats altered by human activity. As in other parts of the world, global climate change also affects Europe, and as it is known, these factors promote the hybridization process and hybrids are, most often, naturally better adapted to changing conditions. *J. erucifolia* is a rare species in Poland and occurs rather in small populations. So far, there are no clear signals that we are dealing with threats caused by hybrids but the fact that admixed genotypes have been observed in both populations defined as pure suggests such possibility. At the same time, we should remember that the process leading to the extinction of pure parental genomes takes time and may go unnoticed for a long time. According to Muhlfeld et al. (2014), “hybridization is an absorbing process, at some point all apparently phenotypically pure individuals of one or both parental lineages may have a hybrid ancestry, leading to the extinction of pure parental genomes”.

On the other hand, hybridization can counter extinction by maintaining or increasing population sizes. This phenomenon is known as the rescue effect of hybridization (Chan et al., 2019; Pfennig et al., 2016) and is stronger when there is an asymmetry in the direction of introgression (backcrossing; Mesgaran et al., 2016). According to Chan et al. (2019), genetic or evolutionary rescue is possible when the population is restored through hybridization. It is worth noting that our research showed a clear asymmetry toward *J. erucifolia* and a significant share of backcrosses in the group of analyzed hybrids. Currently, it is difficult to assess whether the hybridization process can significantly affect *J. erucifolia* populations in Poland, but we hope that the ongoing studies concerning other populations, will allow us to obtain more information. To sum up, this study provides the first molecular confirmation that natural hybrids between *J. vulgaris and J. erucifolia* occur in Poland. Hybridization appeared asymmetrical and bidirectional, with *J. vulgaris* as the usual maternal parent. Nuclear and chloroplast DNA markers were employed to test the hypothesis of natural hybridization. We proved the hybrid nature of all 25 morphologically intermediate individuals. Moreover, we demonstrated that several individuals described as pure species in the field, such as *J. erucifolia* (K3, 6, 8, 10 and S6) and *J. vulgaris* (Pu3, 7, and 9), probably were also of hybrid origin, being backcrosses to *J. erucifolia*) and *J. vulgaris*, respectively.

## AUTHOR CONTRIBUTIONS

**Barbara Gawrońska:** Conceptualization (lead); investigation (equal); methodology (lead); writing – original draft (lead). **Małgorzata Marszałek:** Investigation (equal). **Piotr Kosiński:** Formal analysis (equal); writing – review and editing (equal). **Marek Podsiedlik:** Resources (equal). **Leszek Bednorz:** Resources (equal). **Joanna Zeyland:** Funding acquisition (lead); writing – review and editing (equal).

## CONFLICT OF INTEREST STATEMENT

The authors declare that they have no conflict of interest.

## BENEFIT‐SHARING STATEMENT

In this case, Nagoya Protocol is not applicable, so there are no benefits to report.

## Data Availability

Nuclear DNA and cpDNA sequences have been deposited and are available in the NCBI GenBank. Accession numbers: OP764514–OP764528 and OP806269–OP806272, respectively.
